# Lunatic Asylum of Ohio

**Published:** 1834

**Authors:** 


### II.—Lunatic Asylum of Ohio.

Early in the year 1821, the Editor of this Journal, made a personal application to the General Assembly of Ohio, to establish and endow a state Asylum for the Insane, to be located in Cincinnati. It was successful, and on the 27th of January of that year, a law was passed, instituting an infirmary of that kind, in connexion with a commercial hospital, for boatmen of Ohio, sick at Cincinnati, and a poor-house for the paupers of the city, or, as it was technically called, the township. A considerable sum of money was given by the state for the erection of suitable edifices, the deficiency to be made up by the township; and one half of the auction duties accruing here, were pledged, forever, as an endowment; the poor taxes of the township of Cincinnati, to meet any expenditure beyond the means thus provided by the state. To the insane persons admitted into the Asylum, rich and poor, the professors of the Medical College of Ohio, were to give gratuitous medical assistance. Of this Lunatic Hospital, the associate judges of the court of Common Pleas for this county, and two delegates from the Medical Convention of Ohio, to be chosen annually, under a law since repealed, were appointed inspectors; and it was provided in the act, that if at any time, said judges should report, that, the Trustees of the Township of Cincinnati, had failed to provide comfortable accommodations, and the means of safe keeping, and appropriate medical treatment, it should be in the power of the state to reclaim the money it had granted.

In this way the Legislature of 1820—21, supposed it had made adequate provision for the keeping and cure (if practicable) of such citizens of Ohio as might unfortunately become insane; and humane persons every where in the state, were gratified to think, that the practice of confining those bereft of their reason, in the common jails, would no longer be necessary.

But no expectation could have been more illusory. The building erected by the trustees of the township of Cincinnati, for the safe-keeping, comfortable accommodation, and regular medical treatment of the insane, is most limited in extent, defective in plan, objectionable in location, and inadequate in the means of comfort, custody, and cure. So much so, indeed, that the professors of the Medical College, as they admit, do not pretend to afford professional aid in any case of mental alienation. The grounds about the edifice are scarcely three times as extensive, as the narrow building itself; for a long time, the only promenade for the insane of both sexes was the yard of the poor-house, amidst superannuated debauchees and drunkards; whilst for a time, as we ourselves witnessed, the individual who carried the key of the wards, was a pauper! From the weakness of the grates of the windows, chiefly of wood, many lunatics have been chained to the centre of the floor, to prevent their escape; and several, at different times, have actually gotten out, through the windows or otherwise, and wandered off, even in the depths of winter. One patient, a gentleman of Point-pleasant, Virginia, who was received into the establishment, by agreement with its governors, left it, and returned home on foot, in the midst of winter, over a distance of more than 200 miles; and another, a respectable female of the upper end of our state, escaping, returned to her friends at a still greater distance, in the same season of the year. So bad indeed, is the construction and economy of this, miscalled, asylum, that the friends of a most respectable physician, resident in the interior of Ohio, and affected with insanity, were lately under the necessity of sending him to Philadelphia, for confinement.

All this faithlessness on the part of the township of Cincinnati, has become known to the Legislature of Ohio, not by the report of the inspectors originally appointed, but of two boards of commissioners, one of which visited the institution in 1831, and the other in 1833; still that honorable body has not thought it necessary to act on either of these reports, although they were made at a considerable expense to the state. The latter was embraced in a report on the state of the Medical College of Ohio, and expressly recommended new legislation on the subject of the asylum, but the committee of the senate reported, that no legislation was necessary, and that honorable body, without inquiry or deliberation, adopted the report with but one dissenting voice!

It is time that the medical profession of Ohio, opened their eyes upon this matter. It is, in all respects, a legitimate subject for them to act upon. They are the natural guardians of the public health; they know what means and appliances, both physical and moral, are necessary to the safekeeping and cure of maniacs; they are aware that these can only be had in public institutions; and they have repeatedly been at a loss what advice to give the friends of those who have fallen into insanity. They should relieve themselves from this embarrassment, and support the dignity of their profession, by a simultaneous appeal to the General Assembly; and continue their efforts, till they effect the great work, of procuring for the people of Ohio an infirmary, in which the subjects of mental alienation, every year increasing in number, may be safely lodged, kindly governed, and brought under the most approved plans of medical and social treatment.

We trust that our western readers beyond the limits of Ohio, will not look upon this article with indifference. Except the Lunatic Hospital of Kentucky, at Lexington, which is a well appointed establishment, there is, as far as we are informed, no other infirmary for the insane in the valley of the Mississippi; and for many years, the new states cannot erect them; meanwhile they must rely on those of other states, and are, therefore, interested in the condition of that to which this article is designed to call the attention of the people of Ohio.

